# Synthetic Cannabinoids and ST-Elevated Myocardial Infarction: A Case Report and Literature Review

**DOI:** 10.7759/cureus.39236

**Published:** 2023-05-19

**Authors:** Hassaan Iftikhar, Maryam Saleem, Hafiz S Naeem, Dania Fatima

**Affiliations:** 1 Nephrology, Ohio Valley Nephrology Associates, Owensboro, USA; 2 Internal Medicine, Saint Francis Medical Center, Trenton, USA; 3 Nephrology, Washington University School of Medicine, Saint Louis, USA; 4 Internal Medicine, Waterbury Hospital, Waterbury, USA; 5 Internal Medicine, Nishtar Medical College, Multan, PAK

**Keywords:** marijuana abuse, drug addiction, addiction, cardiac chest pain, k2, synthetic cannabinoids, st elevated myocardial infarction (stemi)

## Abstract

The use of synthetic cannabinoids and marijuana has been known to be associated with myocardial infarction and coronary vasospasms according to a few case reports published for the pediatric population. The data on the use of synthetic cannabinoids and myocardial infarction in adults however is limited. The adverse effects of these so-called designer drugs have been far-reaching. Here, we describe a case of an adult male with ST-elevated myocardial infarction diagnosed secondary to smoking synthetic cannabinoids.

## Introduction

Coronary artery disease is one of the leading causes of morbidity and mortality, and atherosclerosis is the most common underlying cause [1]. Interestingly, in the literature, there have been only a few previously described cases of young adults and adolescents suffering from myocardial ischemia from synthetic cannabinoids; however, data in both pediatric and adult populations is limited [2-4]. Here, we describe a novel case of an adult presenting with ST-elevated myocardial infarction secondary to synthetic cannabinoid (K2) use.

## Case presentation

**Figure 1:**
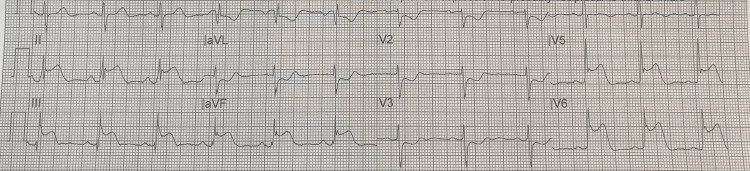
ST elevation in leads II, III, and aVF

The patient was a 43-year-old male who presented to the emergency room as he had severe substernal crushing chest pain which woke him up from sleep and was 10/10 in intensity, sudden in onset, progressively worsening, ongoing for one hour, radiating to the left arm, no associated aggravating factors, and relieved by sublingual nitroglycerine. The patient stated that he had smoked K2 that night for the first time. His past medical history was significant for depression. His family history was significant for premature coronary artery disease in his mother. His social history was significant for smoking marijuana in the past (one stick per day) and K2 use for the first time on the day of presentation to an emergency room. The patient was found to have a significant troponin elevation of 0.64 ng/mL which subsequently trended down to an undetectable level over the course of the next 12 hours. His urine drug screen was negative for cocaine but positive for tetrahydrocannabinol (THC). His low-density lipoprotein (LDL) level was 80 mg/dL. An echocardiogram was performed which revealed no wall motion abnormality, normal left ventricular structure, normal ejection fraction, and normal pericardium. His electrocardiogram (EKG) revealed ST elevation in leads II, III, aVF, V5, and V6 and mirror images in V1-V3 (Figure 1).

He was diagnosed with ST-elevated myocardial infarction (STEMI) and was treated with aspirin 325 mg, clopidogrel 300 mg, and started on systemic heparin drip per hospital protocol for STEMI. The patient had a coronary angiogram performed one hour after presentation to the emergency room which revealed no atherosclerotic lesion or thrombosis. His EKG changes resolved on subsequent EKGs after the coronary angiogram. The patient was diagnosed as having chest pain due to coronary vasospasms. The patient revealed that he was smoking K2, and thus, a link between his coronary vasospasms and chest pain was made. The patient was counseled on cessation of street drug abuse and discharged home with an appointment for outpatient follow-up for longitudinal monitoring.

## Discussion

About 60 different cannabinoids are found in marijuana. Synthetic cannabinoids are artificially manufactured and considered to be a class I controlled substance by the United States Drug Enforcement Agency [5,6], and their use has been on the rise in the past few years.

Synthetic cannabinoids are chemically synthesized by adding compounds to marijuana, can be laced with various substances, and are known by different names such as Spice, Spice X, and Chill X [1,3-7]. Its abuse is common both in Europe and the United States of America. K2 has become a popular alternative to marijuana due to its psychoactive properties, not being detected in routine urine drug screens, and easy availability [2,4]. Acute myocardial infarction (MI) has been known and reported in the literature after the use of marijuana but is not as common after synthetic cannabinoid abuse [4,7]. There have only been a handful of case reports and case series for K2-induced myocardial ischemia and that too in the pediatric population [2,3,8]. Our case is unique in the fact that it has been an adult male for having been diagnosed with ST-elevated myocardial infarction (STEMI) secondary to K2 use. The active compound of cannabis is D9 tetrahydrocannabinol (THC). The pharmacological effect of cannabis involves two receptors, cannabinoid receptor type I (CB1) and cannabinoid receptor type 2 (CB2), which are widely distributed in the cardiovascular system [1]. Synthetic cannabinoid acts on CB1 to produce psychoactive effects [9]. The pathophysiologic effect of marijuana on causing myocardial ischemia is understood to be an increase in sympathetic nervous activity leading to increased heart rate, thus increased myocardial oxygen demand and at the same time increase in carboxyhemoglobin levels leading to a decreased capacity to supply oxygen thus causing myocardial ischemia [2,10]. Of note, synthetic cannabinoids have a higher binding capacity for cannabinoid receptors thus potentiating the side effects [11,12]. The acute intoxication presents as tachycardia, tremors, slurred speech nystagmus, and nausea; however, our case is interesting as chest pain was the only presenting manifestation [13,14]. Although in our case, the patient had a family history of premature coronary artery disease, the diagnosis is generally made clinically based on history and physical exam as laboratory analysis for detecting synthetic cannabinoids is complicated and limited. Most laboratories do not have this testing readily available.

The fact that our patient had only one risk factor for premature coronary artery disease which is family history, but otherwise normal LDL, no diabetes or smoking history, and no underlying coronary atherosclerosis on coronary angiogram, but was found to have ST elevation in inferior and lateral leads with elevated troponin makes it pertinent to note that K2 is linked to causing coronary vasospasms as well as myocardial ischemia with underlying pathophysiology as described above [3]. There have been previous descriptions of adolescents and young adults in the literature with myocardial ischemia secondary to K2 use but very rare to see adults having STEMI from K2 abuse [8].

## Conclusions

K2 also known as spice (synthetic marijuana) is emerging as a scourge in society affecting the both pediatric and adult patient population. This case highlights the importance to obtain social history regarding drug abuse and look beyond cocaine and marijuana, especially in cases with minimal to no risk factors for coronary artery disease and negative drug screen to elicit exposure to K2. The urine and blood drug detection tests need to be made readily available to identify this potentially harmful drug of abuse.
